# Dengue fever: a new challenge for China?

**DOI:** 10.3402/gha.v7.26421

**Published:** 2014-12-18

**Authors:** Chengshen Jiang, John S. Schieffelin, Jian Li, Wenjie Sun

**Affiliations:** 1 Maryland Institute for Applied Environmental Health, University of Maryland, College Park, MD, USA; 2 Department of Pediatrics, Section Adult & Pediatric Infectious Disease, Tulane University School of Medicine, New Orleans, LA, USA; 3 Department of Biostatistics and Bioinformatics, Tulane University School of Public Health and Tropical Medicine, New Orleans, LA, USA; 4 School of Food Science, Guangdong Pharmaceutical University, Zhongshan, China; 5 Department of Global Health and Environmental Sciences, Tulane University School of Public Health and Tropical Medicine, New Orleans, LA, USA

Dengue outbreaks in the Guangdong province reached epidemic proportions in the last quarter of 2014. According to the Guangdong provincial health and family planning commission (1), as of October 27, the total number of dengue fever cases with clinical and laboratory diagnoses reached 41,155. Geographically, more than 80% of dengue cases were reported in Guangzhou City (the capital of the Guangdong province) and its neighbouring prefectures—showing the susceptibility to dengue of areas with dense populations.

Although dengue, one of the most strongly emerging, neglected tropical diseases worldwide and presently without widely available drugs or vaccines, has a geographical distribution in South China (2), this outbreak of dengue fever was the first in South China for almost 20 years. For example, only 120 cases of dengue were reported in China during the year 2011 (2). Of note, the number of imported cases of dengue is on the rise. Moreover, a previous study using spatial scan cluster analyses suggested that counties around Guangzhou City and Chaoshan Region were at increased risk for dengue fever (3). The recent increase in cases could be due to imported dengue cases combined with climatic change (4). In 2009, dengue virus serotype 3 (DENV-3) was first detected in Guangzhou and another isolated strain belonging to genotype II was identified (5). A later investigation reported that three imported cases separately travelled back from Vietnam, India, and Tanzania. The Tanzanian case was confirmed to be the index for the dengue outbreak in Guangdong in 2010 (6). Global climate change has been recognised as a contributor to many infectious diseases (7). Weather factors have been associated with dengue, for example, a previous study used time series Poisson regression analysis on the monthly weather data from Guangzhou and pointed out that minimum temperature and minimum humidity, at a lag of 1 month, were positively associated with dengue incidence in the subtropical city of Guangzhou (8). Guangzhou is close to the tropics and is thus sensitive to the effects of climate change, so an outbreak was probably only a matter of time.

China needs to tackle the increase in dengue. The lessons learnt from previous infectious disease outbreaks such as SARS and H7N9 can be of value. The Chinese government has taken the following surveillance and control measures for dengue: strengthening surveillance and situation analysis; reinforcing case management and medical treatment; and conducting risk communication with the public and releasing information. Of note, the Patriotic Health Campaign Committee Office is a government organisation in each city or county, which can organise the resources needed to address issues of community hygiene. The government should also advise travellers to areas with known dengue outbreaks to take precautions such as avoiding mosquitoes.

*Chengshen Jiang* Maryland Institute for Applied Environmental Health University of Maryland College Park, MD, USA *John S. Schieffelin* Department of Pediatrics Section Adult & Pediatric Infectious Disease Tulane University School of Medicine New Orleans, LA, USA *Jian Li* Department of Biostatistics and Bioinformatics Tulane University School of Public Health and Tropical Medicine New Orleans, LA, USA *Wenjie Sun* School of Food Science Guangdong Pharmaceutical University Zhongshan, China Department of Global Health and Environmental Sciences Tulane University School of Public Health and Tropical Medicine New Orleans, LA, USA Email: wsun3@tulane.edu

